# Risk factors for delayed recovery of liver enzymes in pediatric primary Epstein–Barr virus hepatitis

**DOI:** 10.3389/fped.2026.1934882

**Published:** 2026-10-28

**Authors:** Jiahui Wu, Yuanxi Bian, Fangfang Cheng, Xingyu Li, Jianmei Tian

**Affiliations:** 1 Department of Infectious Diseases, Children’s Hospital of Soochow University, Suzhou, China; 2 School of Pediatric Clinical Medicine, Suzhou Medical College of Soochow University, Suzhou, China

**Keywords:** children, delayed recovery, Epstein–Barr virus, liver enzymes, viral hepatitis

## Abstract

**Objective:**

Children with primary Epstein–Barr virus (EBV) infection frequently develop varying degrees of liver dysfunction. This study aimed to identify clinical risk factors associated with delayed liver enzyme recovery in pediatric primary EBV hepatitis.

**Methods:**

In this retrospective study, 338 children diagnosed with primary EBV hepatitis and admitted to our department between January and December 2024 were enrolled. Based on follow-up liver enzyme recovery, patients were categorized into a normal recovery group and a delayed recovery group. Demographic, clinical, and laboratory data were collected from medical records. Univariate analysis was first performed to identify potential risk factors for delayed recovery, followed by multivariate logistic regression to determine independent predictors. The predictive value of these factors was assessed using receiver operating characteristic (ROC) curve analysis. A risk scoring system was developed based on the regression coefficients.

**Results:**

Among the 338 patients, 228 exhibited normal recovery of liver enzymes, while 110 showed delayed recovery. Patients with delayed recovery had significantly longer fever duration, longer hospital stays, and a higher incidence of hepatomegaly (all *P* < 0.05). Laboratory findings revealed that the delayed recovery group had significantly higher levels of lymphocyte percentage, atypical lymphocyte percentage, aspartate aminotransferase (AST), alanine aminotransferase (ALT), lactate dehydrogenase (LDH), gamma-glutamyl transferase (GGT), alkaline phosphatase (ALP), and ferritin, but significantly lower levels of neutrophil percentage, double-negative T cell percentage, and NK cell percentage compared to the normal recovery group (all *P* < 0.05). Multivariate logistic regression identified fever duration, neutrophil percentage, and ALT level as independent risk factors for delayed recovery. A scoring system was devised assigning 1 point each for fever duration ≥6 days and neutrophil percentage ≤19.0%, and 2 points for ALT ≥118.0 U/L. Using a cutoff score of ≥3 points, this model predicted delayed liver enzyme recovery with 68.20% sensitivity and 73.70% specificity. The area under the ROC curve for this prediction score was 0.77 [95% confidence interval (CI): 0.70–0.84].

**Conclusions:**

Lower neutrophil percentage, higher ALT level, and prolonged fever duration were identified as independent risk factors for delayed recovery of liver enzymes in pediatric primary EBV hepatitis. Clinicians should closely monitor high-risk patients and strengthen outpatient follow-up.

## Introduction

Epstein–Barr virus (EBV) is a herpesvirus for which humans are the exclusive host. Primary infection often occurs during childhood and typically presents with the triad of fever, pharyngitis, and cervical lymphadenopathy (1). In most cases, the infection follows a benign and self-limited course. However, 40%–80% of patients with EBV-induced infectious mononucleosis (IM) develop liver inflammation, primarily presenting as jaundice or acute hepatitis (2–4). Although the majority of patients with EBV-associated hepatitis have a favorable prognosis, rare cases of severe and potentially fatal hepatitis have been reported (5–7).

Children with primary EBV infection frequently develop hepatitis, characterized by mild to moderate elevations in liver transaminases. The underlying pathogenesis and immune mechanisms driving this liver injury remain incompletely understood. Consequently, there is a current lack of specific diagnostic criteria and established treatment guidelines. Identified risk factors for EBV-related hepatitis include older age, prolonged duration of fever, and elevated white blood cell and lymphocyte counts (8, 9). In most patients, serum alanine aminotransferase (ALT) and aspartate aminotransferase (AST) activities become abnormal within the first to third week of illness, typically normalizing by the fourth week (10, 11). However, a subset of patients exhibits delayed recovery, with ALT levels failing to return to normal beyond four weeks of the disease course. Importantly, previous studies have primarily focused on the occurrence or severity of EBV-related liver injury rather than on the subsequent recovery of liver function. The predictors of delayed biochemical recovery after EBV hepatitis therefore remain poorly defined. To our knowledge, no study has specifically investigated delayed liver enzyme recovery in children with primary EBV hepatitis.

We retrospectively evaluated the medical records of children hospitalized with primary EBV hepatitis at our institution. Based on their clinical information and laboratory results, we identified the risk factors for delayed recovery of liver enzymes. This information can guide clinical follow-up strategies for high-risk patients.

## Methods

### Patients and definitions

This retrospective study was approved by the Ethics Committee of Children's Hospital of Soochow University (Approval No. 2025CS183). The study participants were children diagnosed with primary EBV hepatitis, who were hospitalized in the Department of Infectious Diseases at Children's Hospital of Soochow University between January 2024 and December 2024. The inclusion criteria were as follows: (1) Presence of clinical manifestations compatible with IM, such as fever, eyelid swelling, pharyngitis with tonsillar exudate, cervical lymphadenopathy, hepatomegaly, and/or splenomegaly. (2) Serological profile consistent with primary acute EBV infection: positive anti-viral capsid antigen (VCA) IgM, low-affinity VCA IgG, and negative anti-Epstein–Barr nuclear antigen (EBNA) IgG. (3) Diagnosis of primary EBV-associated hepatitis, defined as serum ALT levels above the age-specific reference range (10, 12). (4) Availability of follow-up data for at least six months after hospital discharge. Exclusion criteria were as follows: (1) A history of prior EBV infection. (2) Coinfection with cytomegalovirus (CMV) or any hepatotropic virus. (3) Presence of comorbidities, including chronic liver disease, malignant tumors, or congenital underlying diseases. (4) Incomplete hospitalization data. Flowchart of the study population is shown in Figure 1.

**Figure 1:**
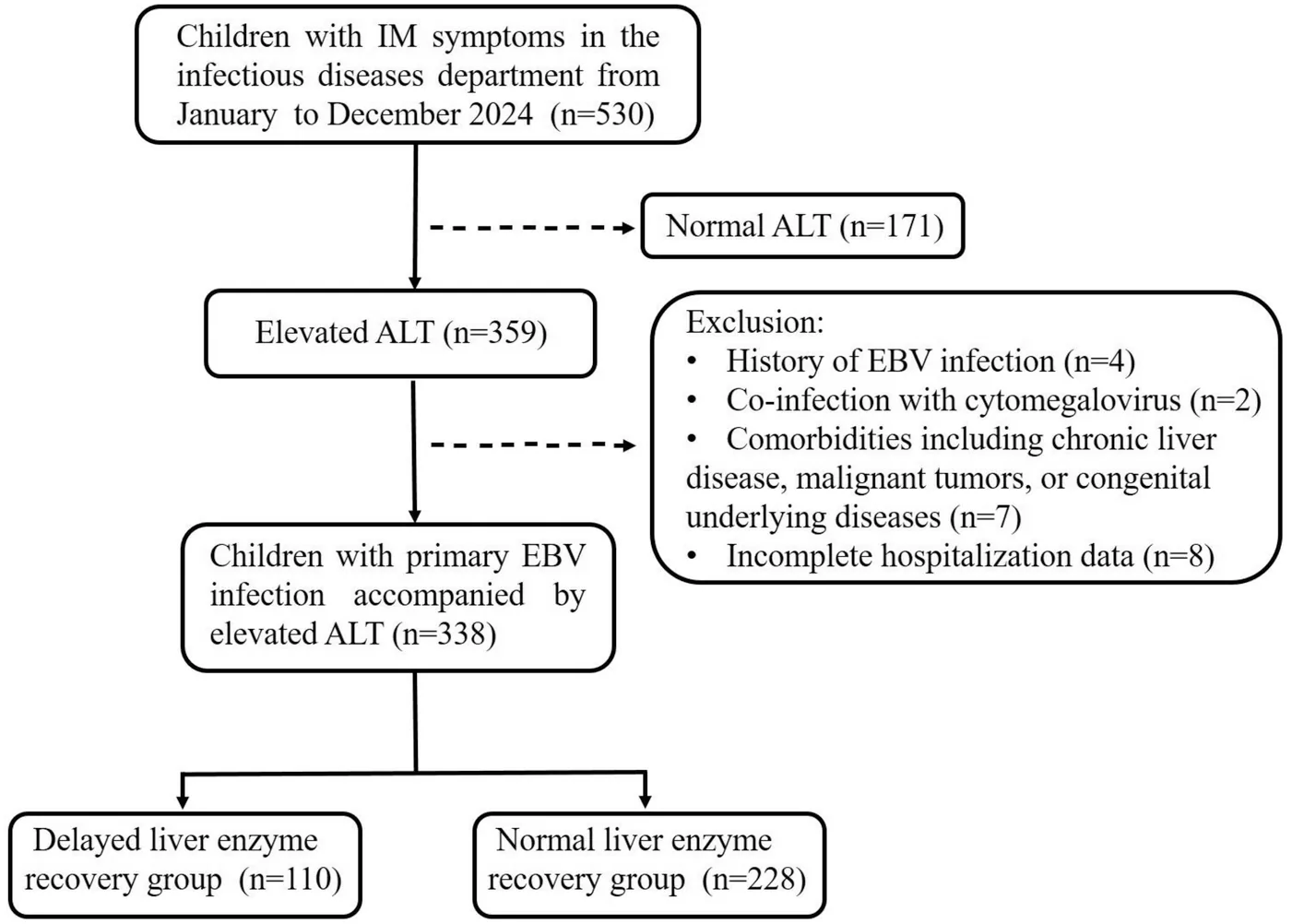
Flowchart of the study population.

For pediatric patients with confirmed primary EBV hepatitis, symptomatic support along with hepatoprotective and enzyme-lowering therapies was the preferred initial management. In our center, the decision to use acyclovir was made by the attending physician based on clinical judgment, and acyclovir was generally used to treat patients with severe or rapidly progressive conditions, such as severe liver involvement (ALT ≥ 5 times the upper limit of normal), prolonged high fever (>7 days), or complications requiring hospitalization. Acyclovir was administered intravenously at a dose of 10 mg/kg every 8 h for 5–7 days. In severe cases complicated by significant upper airway obstruction, myocardial injury, encephalitis, hemolytic anemia, or thrombocytopenia, a short course of glucocorticoids (e.g., methylprednisolone 1 mg/kg/day) was given for 2–3 days. Delayed recovery of liver enzymes was defined as ALT levels remaining above the age-specific reference range beyond 4 weeks of illness onset. Normal recovery was defined as the return of ALT to within the normal range within 4 weeks of illness onset. For patients whose ALT normalized by 4 weeks after illness onset, no routine retesting was performed in the absence of clinical deterioration, and they were followed up via the hospital's internet-based follow-up platform for at least 6 months. For those whose ALT had not normalized by 4 weeks, in-person reassessment was recommended approximately every 2 weeks until complete recovery, with a total follow-up duration of at least 6 months. Reference ranges for liver enzymes were based on *Zhu Futang Practice of Pediatrics* and were listed in Supplementary Table S1 (12).

### Data collection

Demographic, clinical, laboratory, and abdominal ultrasound data were retrospectively collected for all children. Fever duration was defined as the interval from the first onset of fever to the resolution of fever, including both the pre-admission and inpatient periods if fever persisted after admission. Laboratory tests performed within 24 h of admission included complete blood count, biochemical panel (including liver function indices), humoral and cellular immunity markers, ferritin, EBV antibodies, and plasma EBV-DNA copy number. Follow-up liver function data obtained after discharge were used for outcome assessment.

### Statistical analysis

All statistical analyses were performed using SPSS software (version 27.0). Continuous data with non-normal distribution were expressed as medians (interquartile ranges, IQR) and compared using the Mann–Whitney *U* test. Categorical data were presented as numbers (percentages) and analyzed using the Pearson's chi-square test or Fisher's exact test. A two-tailed *P* value <0.05 was considered statistically significant. Variables identified as significant in univariate analysis were entered into a multivariate logistic regression model to identify independent risk factors for delayed recovery of liver enzymes. Continuous variables were converted into dichotomous variables based on the optimal cutoff values determined by the Youden index from receiver operating characteristic (ROC) curves for each independent predictor. The Hosmer–Lemeshow test was used to assess the goodness-of-fit of the regression model. The predictive performance of each independent risk factor was evaluated using the area under the ROC curve (AUC). The regression coefficients (*β*) from the final multivariable logistic regression model were used to develop the scoring system. The *β* coefficient of each independent predictor was divided by the smallest *β* coefficient and rounded to the nearest integer to assign points.

## Results

### Clinical characteristics

A total of 530 children with symptoms of IM were initially screened in the infectious diseases department between January and December 2024. Of these, 171 had normal ALT levels and were excluded. Among the remaining 359 children with elevated ALT, 21 were further excluded: 4 with a history of prior EBV infection, 2 with CMV co-infection, 7 with comorbidities (chronic liver disease, malignant tumors, or congenital underlying diseases), and 8 with incomplete hospitalization data. Finally, 338 children with primary EBV hepatitis met the inclusion criteria and were regularly followed up (Figure 1). Table 1 presents the demographic and clinical characteristics of the patients. The cohort comprised 110 cases (32.54%) in the delayed recovery group (ALT persistently above the age-specific normal range for >4 weeks after illness onset) and 228 cases (67.46%) in the normal recovery group (ALT normalized within 4 weeks after illness onset). Male patients accounted for 40.91% (45/110) and 45.18% (103/228) of the delayed recovery and normal recovery groups, respectively. The median ages were similar between the two groups (delayed recovery group: 6.33 years; normal recovery group: 6.50 years). In terms of clinical manifestations, the duration of fever was significantly longer in the delayed recovery group than in the normal recovery group, and the incidence of hepatomegaly was also significantly higher. Regarding antiviral and glucocorticoid therapy, no significant differences were observed between the two groups. However, the length of hospital stay was significantly longer in the delayed recovery group.

**Table 1:** Clinical characteristics of children with primary EBV hepatitis in normal and delayed recovery groups .

Variables	Normal recovery group	Delayed recovery group	Statistic	*P* value
Age in years, median(IQR)	6.50 (4.71–8.21)	6.33 (4.75–8.67)	−0.30	0.765
Male, n(%)	103 (45.18%)	45 (40.91%)	0.55	0.459
Fever, *n* (%)	209 (91.67%)	103 (93.64%)	0.41	0.524
Fever duration in days	5.00 (3.50–7.00)	7.00 (4.50–10.00)	−3.09	0.002
Eyelid swelling, *n* (%)	154 (67.54%)	83 (75.45%)	2.22	0.137
Pharyngitis with tonsillar exudate, *n* (%)	187 (82.02%)	86 (78.18%)	0.70	0.402
Cervical lymphadenopathy, *n* (%)	226 (99.12%)	110 (100.00%)	/	1.000
Hepatomegaly, *n* (%)	85 (37.28%)	58 (52.73%)	7.25	0.007
Splenomegaly, *n* (%)	116 (50.88%)	66 (60.00%)	2.49	0.115
Antiviral drugs usage, *n* (%)	117 (51.32%)	67 (60.91%)	2.75	0.097
Glucocorticoids usage, *n* (%)	23 (10.09%)	12 (10.91%)	0.05	0.816
Hospitalization days	7.00 (6.00–9.00)	8.00 (7.00–10.00)	−3.32	<0.001

EBV, Epstein–Barr virus, IQR, interquartile range. Antiviral therapy: intravenous acyclovir.

### Laboratory findings

All laboratory values presented in Table 2 were obtained within 24 h of admission, and the median time from symptom onset to admission was 5.5 days (IQR: 3–7.5). The laboratory findings for the normal and delayed recovery groups are summarized in Table 2. In the peripheral blood differential, the delayed recovery group had a significantly higher percentage of lymphocytes and atypical lymphocytes, but a significantly lower percentage of neutrophils, compared with the normal recovery group. For biochemical profiles, the delayed recovery group showed significantly higher levels of AST, ALT, lactate dehydrogenase (LDH), gamma-glutamyl transferase (GGT), and alkaline phosphatase (ALP) than the normal recovery group. In terms of cellular immunity, the percentages of double-negative T cells and natural killer (NK) cells were lower in the delayed recovery group. Additionally, ferritin levels were significantly elevated in this group. However, no significant differences were observed between the two groups in humoral immunity or plasma EBV-DNA copy number.

**Table 2:** Laboratory values of children with primary EBV hepatitis in normal and delayed recovery groups .

Variables	Normal recovery group	Delayed recovery group	Statistic	*P* value
Blood routine results, median(IQR)				
WBC, ×10^9^/L	15.02 (11.95–18.58)	15.21 (12.62–18.31)	−0.41	0.684
% neutrophils	22.10 (15.95–28.00)	17.40 (12.20–21.05)	−4.04	<0.001
% lymphocytes	71.70 (65.05–77.75)	75.60 (70.60–80.20)	−2.75	0.006
Lymphocyte count, ×10^9^/L	10.34 (8.40–13.99)	11.63 (8.99–14.60)	−1.32	0.188
% atypical lymphocytes	6.00 (4.00–9.50)	8.00 (5.50–12.50)	−2.56	0.011
Hemoglobin, g/L	127.00 (118.00–132.50)	124.00 (120.00–130.50)	−0.46	0.646
Platelet, ×10^9^/L	195.00 (153.00–228.00)	184.00 (143.50–230.50)	−0.50	0.614
CRP, mg/L	6.57 (3.73–11.75)	6.14 (3.98–11.61)	−0.20	0.839
Biochemical results				
AST, U/L	84.90 (60.30–137.20)	146.20 (91.85–218.60)	−4.79	<0.001
ALT, U/L	106.50 (63.80–171.80)	185.30 (122.40–330.45)	−4.69	<0.001
LDH, U/L	551.90 (471.45–649.25)	604.10 (517.25–677.65)	−2.58	0.010
TBIL, μmol/L	6.30 (5.00–8.40)	6.70 (5.40–8.70)	−0.85	0.398
GGT, U/L	48.00 (20.90–97.95)	78.40 (47.15–129.90)	−3.17	0.002
ALP, U/L	242.00 (199.00–308.45)	280.20 (228.10–394.05)	−3.33	<0.001
Humoral immunity results				
IgA, g/L	1.82 (1.43–2.39)	1.88 (1.52–2.52)	−0.50	0.614
IgM, g/L	1.83 (1.41–2.30)	2.01 (1.58–2.47)	−1.43	0.154
IgG, g/L	13.27 (10.72–16.28)	13.04 (11.23–17.14)	−0.26	0.795
Cellular immune results				
CD3 + T, %	85.76 (80.57–88.88)	87.65 (82.94–90.83)	−1.85	0.064
CD3 + CD4 + T, %	12.68 (9.18–16.07)	13.18 (9.91–15.71)	−0.34	0.732
CD3 + CD8 + T, %	65.30 (57.85–69.79)	68.29 (60.34–73.59)	−1.91	0.056
CD4+/CD8+	0.21 (0.14–0.30)	0.17 (0.14–0.24)	−1.66	0.097
CD3 + CD4-CD8-T, %	6.55 (4.34–9.15)	5.16 (2.65–8.46)	−2.24	0.025
CD3-CD19 + B, %	3.47 (2.06–4.75)	2.66 (1.45–4.61)	−1.76	0.078
CD3-CD56+/16 + NK, %	9.78 (7.29–14.09)	8.43 (6.78–12.08)	−2.00	0.046
Ferritin, ug/L	235.00 (131.35–324.20)	272.60 (170.15–440.10)	−2.34	0.019
EBV-DNA copy numbers in plasma, copies/mL	1,630.00 (565.00–3,515.00)	1,950.00(781.50–7,055.00)	−1.25	0.211

EBV, Epstein–Barr virus, IQR, interquartile range, WBC, white blood cell, CRP, C-reactive protein, AST, aspartate aminotransferase, ALT, alanine aminotransferase, LDH, lactate dehydrogenase, TBIL, total bilirubin, GGT, gamma-glutamyl transferase, ALP, alkaline phosphatase.

All laboratory values presented in Table 2 were obtained within 24 h of admission.

### Predictive risk factors for delayed recovery of liver enzymes

A total of 13 clinical and laboratory variables (fever duration, hepatomegaly, neutrophil percentage, lymphocyte percentage, atypical lymphocyte percentage, ALT, AST, LDH, GGT, ALP, double-negative T cell percentage, NK cell percentage, and ferritin) were entered into a logistic regression analysis (Tables 1, 2). Fever duration, neutrophil percentage, and ALT were identified as independent risk factors for delayed recovery of liver enzymes in children with primary EBV hepatitis (Supplementary Table S2). ROC curve analysis with the Youden index was then used to determine optimal cutoff values for these continuous predictors. The optimal thresholds were 6 days for fever duration, 19.0% for neutrophil percentage, and 118.0 U/L for ALT. All three indicators remained statistically significant in the logistic regression model (Table 3). The Hosmer-Lemeshow test yielded a *P*-value of 0.964, indicating excellent calibration between predicted and observed outcomes. The predictive performance of each individual risk factor was assessed by ROC analysis (Figure 2), with AUC values of 0.63 for fever duration ≥6 days, 0.66 for neutrophil percentage ≤19.0%, and 0.67 for ALT ≥ 118.0 U/L. Based on the *β* coefficients of the significant predictors, a scoring system was developed: one point was assigned for fever duration ≥6 days and for neutrophil percentage ≤19.0%, and two points were assigned for ALT ≥118.0 U/L (Table 3). Using a cutoff total score of ≥3 points, delayed recovery of liver enzymes was identified with a sensitivity of 68.20% and a specificity of 73.70%. The AUC for this prediction score was 0.77 (95% CI: 0.70–0.84) (Figure 2). Patients were subsequently stratified into two risk groups based on their total score: a high-risk group (3–4 points) and a low-risk group (0–2 points). The rate of delayed recovery progressively increased with higher total scores (Figure 3).

**Table 3:** Multivariable logistic regression analysis for predicting delayed recovery of liver enzymes in patients with primary EBV hepatitis.

Independent variables	Logistic coefficient(*β*)	OR (95%CI)	*P* value	Score
Fever duration ≥6 days	0.93	2.55 (1.30–4.99)	0.007	1
% neutrophils ≤19.0%	1.06	2.90 (1.48–5.66)	0.002	1
ALT level≥118.0 U/L	1.41	4.09 (2.01–8.29)	<0.001	2

EBV, Epstein–Barr virus, OR, odds ratio, CI, confidence interval.

**Figure 2:**
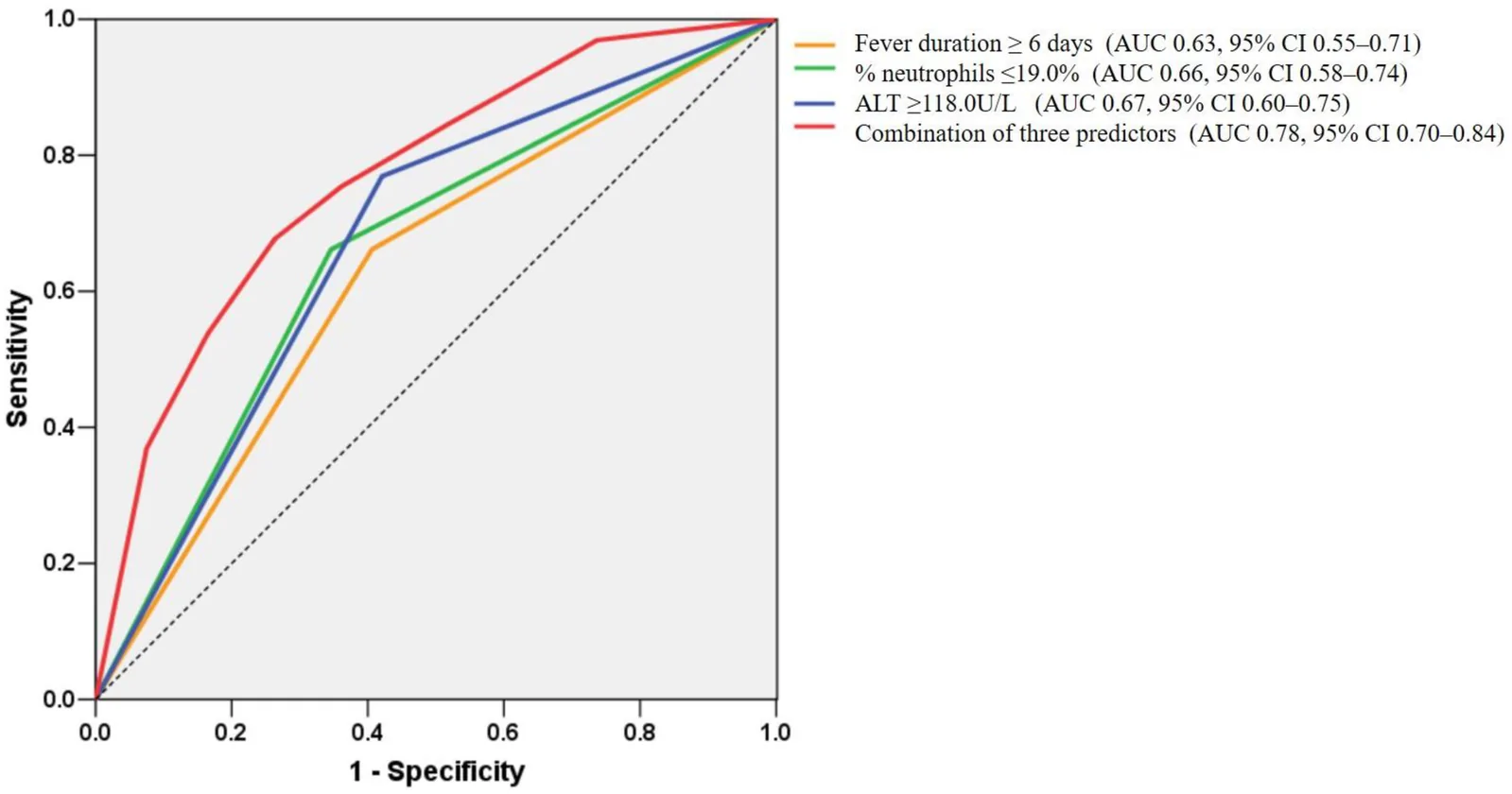
Receiver operating characteristic (ROC) curves of different predictors for delayed recovery of liver enzymes in children with primary EBV hepatitis.

**Figure 3:**
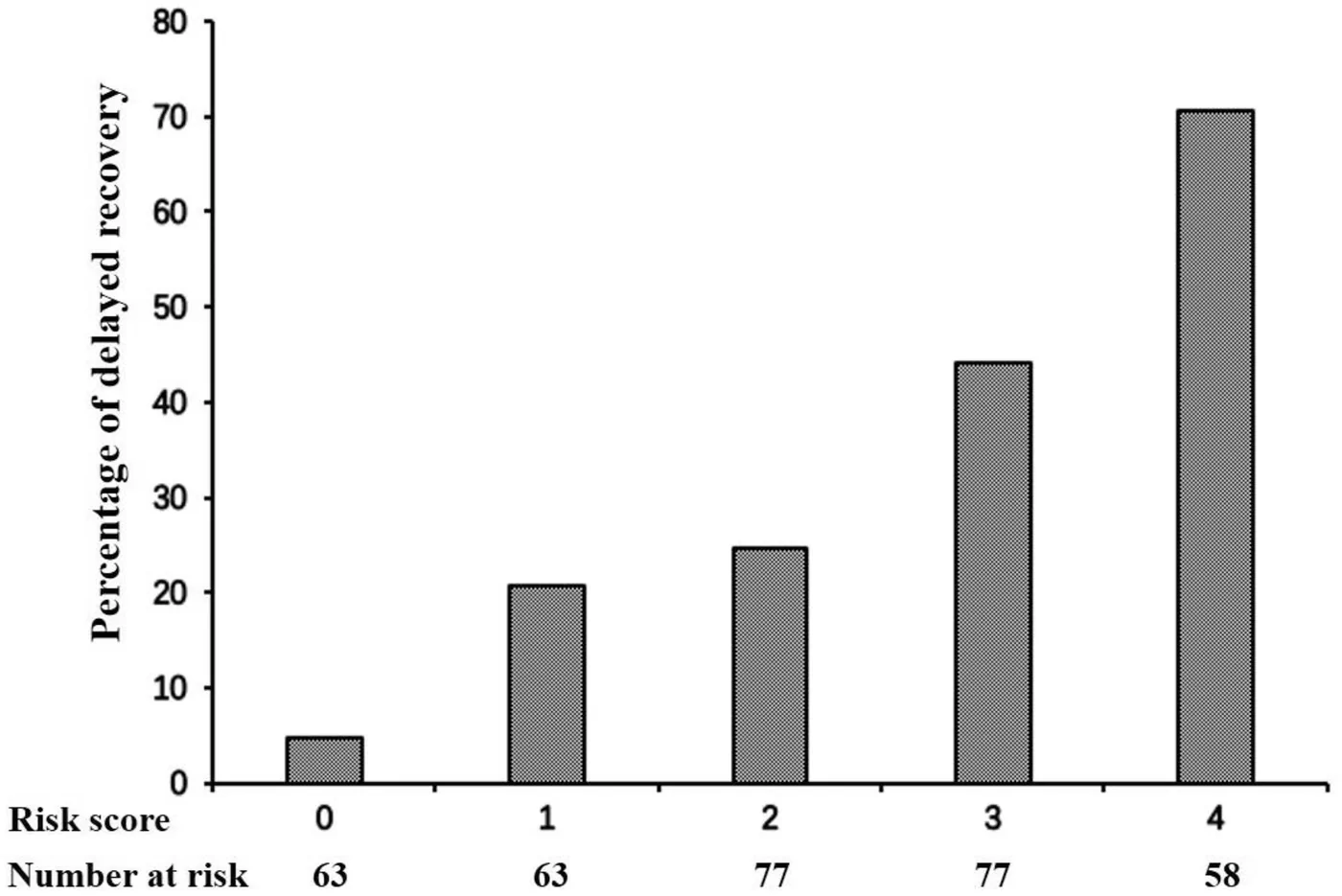
Simple scoring model evaluated for correspondence with delayed recovery of liver enzymes.

## Discussion

EBV hepatitis is typically diagnosed clinically, usually based on the presence of atypical lymphocytosis, elevated liver enzymes, and positive EBV-specific serology (13). Existing literature indicates that EBV hepatitis usually presents with mild, transient transaminase elevation that resolves spontaneously within weeks (14, 15). However, for preschool and school-age children undergoing routine vaccination, delayed liver enzyme recovery may be a clinical concern that could influence decisions about immunization timing, although evidence supporting postponement of vaccination solely due to persistent ALT elevation is lacking. Focusing on this population with prolonged hepatic injury, we conducted a study to identify risk factors for delayed recovery. By analyzing demographic, clinical, and laboratory data, we aimed to develop a scoring system to predict this outcome.

The 4-week cutoff for defining delayed liver enzyme recovery was selected based on the typical biochemical course of EBV hepatitis in children. In a retrospective cohort of 294 children with primary EBV infection and hepatic involvement, the median time to ALT normalization was 20 days (11). Another pediatric study showed that approximately half of the patients achieved normalization of elevated transaminases by 3 weeks (10). Although complete biochemical recovery may take longer in some children, the available pediatric evidence indicates that the median time to ALT normalization is approximately 3–4 weeks. Therefore, 4 weeks represents a clinically meaningful early milestone: if ALT remains above the age-specific normal range beyond this point, the child is considered to have delayed recovery and may require closer follow-up and more intensive monitoring. In our cohort, 32.5% (110/338) of patients had not achieved ALT normalization by 4 weeks after disease onset, a subgroup that might be easily overlooked without regular follow-up. During EBV infection, transaminase elevation is usually less than five times the upper limit of normal (14). In our delayed recovery group, the median ALT and AST levels were 185.30 U/L and 146.20 U/L, respectively, with nearly half of the patients exhibiting elevations exceeding five times the normal limit. Although cholestasis without jaundice has been reported in EBV infection, jaundiced presentations are rare (16, 17). In our delayed recovery group, ALP and GGT levels were significantly higher than those in the normal recovery group, whereas no significant difference in bilirubin levels was observed between the two groups. These findings may suggest a cholestatic component or tendency in patients with delayed liver enzyme recovery, even in the absence of overt hyperbilirubinemia.

In our cohort, 54.4% of patients received acyclovir therapy, which was consistent with the high rates of antiviral use reported in other pediatric inpatient cohorts (18, 19). This practice may have reflected the fact that hospitalized children represented a more severe subgroup, and clinicians may have opted for antiviral therapy when rapid disease progression or severe liver involvement was present. Existing evidence does not demonstrate that acyclovir improves clinical outcomes in immunocompetent children with EBV infection (20). It may only reduce viral replication and oropharyngeal shedding and does not appear to shorten symptom duration or reduce complications (21). Therefore, the high rate of antiviral use in our cohort should be interpreted as reflecting real-world clinical practice rather than evidence-based recommendations. Further studies are needed to clarify the role of antiviral therapy in children with EBV hepatitis.

The liver damage induced by EBV infection is thought to be largely immune-mediated rather than a direct cytopathic effect of the virus (3, 22). During primary EBV infection, B cells are the first target, processing and presenting EBV antigens to T cells. This recognition activates CD8+ cytotoxic T lymphocytes, which subsequently induce apoptosis of infected B cells (23, 24). In our study, the delayed recovery group exhibited significantly lower proportions of double-negative T cells and NK cells, along with significantly elevated ferritin levels, compared to the normal recovery group. However, no significant difference in EBV-DNA load was observed between the two groups. These findings may suggest that an excessive systemic inflammatory response, potentially related to immune dysregulation, may be involved in delayed liver enzyme recovery. However, given the retrospective design, we cannot establish a causal relationship between immune dysregulation and delayed recovery. Whether more active anti-inflammatory interventions could benefit high-risk patients with delayed liver enzyme recovery remains an open question that requires dedicated prospective studies. Bian et al. reported that a reduction in double-negative T cells is an independent risk factor for EBV reactivation in children undergoing hematopoietic stem cell transplantation (25). Additionally, downregulation of NK cell activity reduces the cytotoxicity against EBV-infected cells, representing a mechanism by which EBV evades immune surveillance following infection (26). Further studies are warranted to elucidate the specific immune mechanisms underlying primary EBV hepatitis and delayed enzymatic recovery.

Previous studies have focused on risk factors for the development or severity of EBV-related liver injury (8, 27, 28). However, these studies did not examine the subsequent recovery of liver function after liver injury had occurred. In contrast, the present study addressed a distinct clinical question: which children with primary EBV hepatitis fail to achieve timely normalization of liver enzymes. We identified lower neutrophil percentage (≤19.0%), higher ALT level (≥118.0 U/L), and prolonged fever duration (≥6 days) as independent risk factors for delayed enzymatic recovery. To our knowledge, this is the first study to specifically investigate predictors of delayed biochemical recovery in primary EBV hepatitis, shifting the focus from the occurrence of liver injury to its resolution. In the diagnosis of IM, more attention is typically given to the proportions of peripheral blood lymphocytes and atypical lymphocytes. However, a reduced neutrophil percentage holds greater significance in predicting delayed liver enzyme recovery. This reduction may, on the one hand, be a concomitant phenomenon of the intense T-cell immune response induced by EBV. On the other hand, over activated cytotoxic T cells and Th1-type cytokines (such as IFN-γ and TNF-α) may suppress bone marrow granulopoiesis and exacerbate intrahepatic immune-mediated injury. Neutropenia may reflect a more severe systemic inflammatory state, leading to persistently elevated transaminases and prolonged fever, which in turn necessitates a longer recovery time for affected children. Consistently, previous literature have reported that neutropenia is associated with poor prognosis in EBV-associated hemophagocytic lymphohistiocytosis (EBV-HLH) or severe infectious mononucleosis (29, 30). We developed a scoring system based on the above three independent predictors, weighted by their *β* coefficients: fever duration ≥6 days (1 point), neutrophil percentage ≤19.0% (1 point), and ALT ≥118.0 U/L (2 points). A total score of ≥3 points was defined as indicating a high risk for delayed recovery from liver injury, with a sensitivity of 68.20% and a specificity of 73.70%. For this high-risk population, enhanced outpatient follow-up is warranted. However, vaccination was not evaluated in this study, and whether vaccination should be deferred in these patients remains an untested hypothesis that requires dedicated studies on vaccine safety in this specific population.

This study has several limitations. First, as a single-center, retrospective study that only included hospitalized pediatric patients, our findings may be subject to selection bias. Hospitalized children with EBV infection may represent a more severe subgroup than those managed in community-based or outpatient settings. Therefore, the predictive performance of the identified factors may not be directly applicable to milder EBV infections, which limits the generalizability of our findings to the broader pediatric EBV population. Second, the cut-offs for ALT, neutrophil percentage, and fever duration were derived from the same dataset used to develop the model, which may have led to overfitting and optimistic estimates of predictive performance. To address these limitations, future large-scale, multicenter, prospective cohort studies enrolling both inpatient and outpatient children are warranted to externally validate our scoring system. In addition, dynamic monitoring of cytokine profiles and lymphocyte subsets should be incorporated to elucidate the underlying mechanisms of immune dysregulation.

## Conclusions

In summary, this study identified that neutrophil percentage, ALT level, and fever duration are independent risk factors for delayed recovery of liver enzymes in primary EBV hepatitis. By integrating these independent predictors into a practical scoring system, clinicians can achieve early risk stratification and establish standardized outpatient follow-up schedules for high-risk patients.

## Data Availability

The raw data supporting the conclusions of this article will be made available by the authors, without undue reservation.
