# Supplementary material for: Risk factors for delayed recovery of liver enzymes in pediatric primary Epstein–Barr virus hepatitis
Source: Front Pediatr. 2026 Oct 28;14:1934882. doi: 10.3389/fped.2026.1934882 (PMC13751982; doi:10.3389/fped.2026.1934882)
Supplement: Supplementary file 1 [file Table1.docx]

**Supplementary Table S1** Age- and sex-specific reference ranges for serum ALT, AST, GGT, and ALP.

| Liver enzyme | Age group | Sex | Reference range (U/L) |
| --- | --- | --- | --- |
| ALT | 0-28 days | Both | 5-40 |
| ALT | 28 days-1 year | Both | 8-71 |
| ALT | 1-2 years | Both | 8-42 |
| ALT | 2-13 years | Both | 7-30 |
| ALT | 13-18 years | Male | 7-43 |
| ALT | 13-18 years | Female | 6-29 |
| AST | 0-28 days | Both | 15-70 |
| AST | 28 days-1 year | Both | 21-80 |
| AST | 1-2 years | Both | 22-59 |
| AST | 2-13 years | Both | 14-44 |
| AST | 13-18 years | Male | 12-37 |
| AST | 13-18 years | Female | 10-31 |
| GGT | 0-6 months | Both | 9-150 |
| GGT | 6 months-1 year | Both | 6-31 |
| GGT | 1-13years | Both | 5-19 |
| GGT | 13-18 years | Male | 8-40 |
| GGT | 13-18 years | Female | 6-26 |
| ALP | 0-6 months | Both | 98-532 |
| ALP | 6 months-1 year | Both | 106-420 |
| ALP | 1-2 years | Both | 128-432 |
| ALP | 2-9 years | Both | 143-406 |
| ALP | 9-12 years | Both | 146-500 |
| ALP | 12-14 years | Male | 160-610 |
| ALP | 12-14 years | Female | 81-454 |
| ALP | 14-15 years | Male | 82-603 |
| ALP | 14-15 years | Female | 63-327 |
| ALP | 15-17 years | Male | 64-443 |
| ALP | 15-17 years | Female | 52-215 |
| ALP | 17-18 years | Male | 51-202 |
| ALP | 17-18 years | Female | 43-130 |

*ALT* alanine aminotransferase, *AST* aspartate aminotransferase, *GGT* gamma-glutamyl transferase, *ALP* alkaline phosphatase.

**Supplementary Table S2** Complete multivariable logistic regression analysis of factors associated with delayed liver enzyme recovery in children with primary EBV hepatitis.

| variables | Logistic coefficient(β) | OR (95%CI) | *P* value |
| --- | --- | --- | --- |
| Fever duration in days | 0.12 | 1.13(1.02-1.25) | 0.015 |
| Hepatomegaly | 0.52 | 1.69(0.83-3.45) | 0.152 |
| % neutrophils | -0.11 | 0.88(0.81-0.98) | 0.016 |
| % lymphocytes | -0.07 | 0.93(0.86-1.01) | 0.080 |
| % atypical lymphocytes | 0.03 | 1.03(0.97-1.10) | 0.362 |
| AST | 0.01 | 1.00(0.99-1.01) | 0.728 |
| ALT | 0.40 | 1.35(1.21-1.43) | 0.005 |
| LDH | 0.00 | 1.00(0.99-1.00) | 0.839 |
| GGT | 0.00 | 1.00(0.99-1.01) | 0.982 |
| ALP | 0.00 | 1.00(0.99-1.01) | 0.485 |
| CD3+CD4-CD8-T | -0.08 | 0.93(0.85-1.01) | 0.096 |
| CD3-CD56+/16+NK | -0.05 | 0.95(0.89-1.02) | 0.138 |
| Ferritin | 0.00 | 1.00(0.99-1.00) | 0.720 |

*OR* odds ratio, *CI* confidence interval, *EBV* Epstein–Barr virus, *AST* aspartate aminotransferase, *ALT* alanine aminotransferase, *LDH* lactate dehydrogenase, *GGT* gamma-glutamyl transferase, *ALP* alkaline phosphatase
